# Two decades of diagnostic safety research: advances, challenges, and next steps

**DOI:** 10.1515/dx-2025-0124

**Published:** 2025-10-07

**Authors:** Sundas Khan, Andrea Bradford, Christina L. Cifra, Hardeep Singh

**Affiliations:** Center for Innovations in Quality, Effectiveness and Safety (IQuESt), Michael E. DeBakey Veterans Affairs Medical Center (MEDVAMC), Houston, TX, USA; Department of Medicine, Baylor College of Medicine, Houston, TX, USA; Division of Medical Critical Care, Department of Pediatrics, Boston Children’s Hospital, Harvard Medical School, Boston, MA, USA

**Keywords:** diagnostic safety, diagnostic errors, diagnostic process, patient safety, quality improvement

## Abstract

Since the National Academies of Sciences, Engineering, and Medicine (NASEM) report *Improving Diagnosis in Health Care*, various research efforts have accelerated progress to understand and improve diagnostic safety. In this opinion piece, we summarize two decades of progress in methods for identifying and learning from diagnostic errors and provide recommendations for future research. Multiple methods have been used to quantify diagnostic errors in various clinical settings, thereby facilitating a deeper understanding of the nature and magnitude of the problem and enabling studies of contributing factors. However, the use of standardized definitions of a diagnostic error and/or diagnostic safety event, a shared mental model for measurement, and more universal application of tools to measure these events across the research enterprise are still needed. We highlight progress in selected research methods and applications, such as co-development with patients, inclusion of multidisciplinary perspectives (such as those from informatics, human factors, and social and cognitive sciences), and the use of sociotechnical approaches. Specific areas where research should be prioritized include the application of cognitive science to the real-world study of diagnostic errors, understanding the costs associated with diagnostic safety, developing and implementing interventions related to patient engagement, evaluating and integrating artificial intelligence, and implementing system-related interventions to improve diagnosis. To promote broad-scale improvement in diagnostic safety over the next decade, we provide several actionable steps and recommendations for various audiences, including researchers, research funders, safety professionals, and policymakers, involved in research and implementation activities for reducing preventable diagnostic harm.

## Introduction

Over the past two decades, research has consistently shown that diagnostic errors are a significant concern in every healthcare setting [1]. Epidemiologic studies show that diagnostic errors nearly always have multifaceted causes and arise from an interplay of various contributing factors, including issues related to cognition (such as inadequate data gathering, data interpretation, or clinical assessment), systems, patient factors, and communication [2], [3]. Rates of diagnostic errors in acute care, ambulatory care, and emergency care remain unacceptably high [4].

Since the National Academies of Sciences, Engineering, and Medicine (NASEM) report *Improving Diagnosis in Health Care,* [5] research efforts have accelerated progress to address diagnostic safety. Diagnostic safety research remains crucial to understanding the complexity of the diagnostic process and to inform strategies for improving practice and policy [5]. In this opinion piece, we summarize major progress in methods to identify and learn from diagnostic errors and in understanding and achieving diagnostic safety. We highlight existing knowledge gaps and provide recommendations to accelerate research efforts and their impact on diagnostic safety over the next decade.

## Methodological advances to identify and learn from diagnostic errors

Diagnostic errors have been quantified through multiple methodologies, helping create a better understanding of the nature and magnitude of the problem [6] and enabling studies of contributing factors [7], [8]. The ability to measure diagnostic errors is also essential for monitoring outcomes of interventions to prevent or mitigate these errors.

### Conceptualizing and defining diagnostic errors

Measurement of diagnostic error requires a reliable definition with face validity and usability across people, systems, and contexts. No single definition of diagnostic error has emerged as universal or sufficient to meet the needs of the field. Although this conceptual pluralism makes it difficult to compare diagnostic error rates across studies, the emergence of new definitions has highlighted novel facets of the problem to be measured and understood.

While the groundwork for defining diagnostic errors was laid more than two decades ago, it has been evolving since. Graber and colleagues defined the scope of diagnostic errors as inclusive of missed, delayed, and wrong diagnoses, determined in hindsight [9]. Further, Schiff and colleagues critiqued prior analyses of diagnostic errors for, among other things, a lack of attention to root causes and underlying system vulnerabilities [10]. This approach to analyzing diagnostic errors as *process failures* shifted the focus away from outcomes alone (e.g., accurate vs. inaccurate, harmful vs. benign). These and other conceptual shifts have had a marked influence on subsequent definitions and frameworks for studying diagnostic error. For instance, Singh et al.’s characterization of diagnostic errors as “missed opportunities” emphasized the importance of preventability [11]. NASEM’s definition of diagnostic error expanded most prior definitions by explicitly including failure to communicate the diagnosis to the patient [5]. Most recently, the Agency for Healthcare Research and Quality (AHRQ) proposed a definition of “diagnostic safety event” that combines several of these concepts [12]. Table 1 summarizes widely used definitions and the measurement methods that reflect these different conceptualizations.

**Table 1:** Overview of definitions.

Term	Definition	Measurement methods	Defined by
Diagnostic error	A diagnosis that was unintentionally delayed (sufficient information was available earlier), wrong (another diagnosis was made before the correct one), or missed (no diagnosis was ever made), as judged from the eventual appreciation of more definitive information	Chart review [9]	Graber et al. [9]
Diagnostic error	Any mistake or failure in the diagnostic process leading to a misdiagnosis, a missed diagnosis, or a delayed diagnosis. This could include any failure in timely access to care; elicitation or interpretation of symptoms, signs, or laboratory results; formulation and weighing of differential diagnosis; and timely follow-up and specialty referral or evaluation.	Chart review + DEER taxonomy [10]	Schiff et al. [10]
Diagnostic error	Missed opportunities to make a correct or timely diagnosis based on the available evidence, regardless of patient harm	Chart review + revised safer Dx instrument [13]	Singh et al. [11]
Diagnostic error	The failure to: (a) establish an accurate and timely explanation of the patient’s health problem(s) or (b) communicate that explanation to the patient	Malpractice claims, chart review, root cause analysis, patient or provider reports [14]	NASEM report [5]
Diagnostic safety event	One or both of the following occurred, whether or not the patient was harmed: – Delayed, wrong, or missed diagnosis: There were one or more missed opportunities to pursue or identify an accurate and timely diagnosis (or other explanation) of the patient’s health problem(s) based on the information that existed at the time. – Diagnosis not communicated to patient: An accurate diagnosis (or other explanation) of the patient’s health problem(s) was available, but it was not communicated to the patient (includes patient’s representative or family as applicable).	Common formats for event reporting – Diagnostic safety [12]	AHRQ [12]

### Methods to classify breakdowns and errors in the diagnostic process

Early studies of diagnostic error relied on an operational definition and chart review. While chart review procedures can be standardized within a study, chart review approaches between studies vary. A significant advance has been the development of standardized tools that align with formal definitions of diagnostic error and enable a thorough and replicable approach to reviewing patient information. These brief tools are easily disseminated and can be used in a variety of clinical settings. Examples of standardized tools from Table 1 include:

– Diagnosis Error Evaluation and Research (DEER) taxonomy [10]: a widely used approach for evaluating diagnostic errors. The taxonomy outlines different stages in the diagnostic process where an error may occur, including: (1) access and presentation, (2) history taking/collection, (3) physical exam, (4) testing, (5) assessment, (6) referral, and (7) follow-up.
– Revised Safer Dx Instrument [13]: a 13-item instrument to aid in detecting the presence or absence of diagnostic errors through a structured evaluation of the diagnostic process. Each item describes an aspect of the diagnostic process and is rated from 1 (strongly disagree) to 7 (strongly agree). Items 1–12 are unscored but are used to inform a global impression on the final item, which determines whether a diagnostic error occurred.
– Common Formats for Event Reporting for Diagnostic Safety (CFER-DS) [12]: a reporting format intended to collect meaningful data about diagnostic safety events that can be aggregated and analyzed for learning and improvement at the local, regional, and national levels. CFER-DS content incorporates structure (e.g., type of facility or unit where the event occurred), process (e.g., process breakdowns), and outcomes (e.g., harm type and severity).

Despite the advantages of these standardized tools, chart review is time-consuming and requires clinical competency in diagnosis. Consequently, use of these tools remains limited mainly to research and academic settings, and they have not been universally used in practice or adopted broadly across clinical settings.

### Identifying diagnostic errors in practice

Diagnostic errors are difficult to observe in real time, and measurement is limited by under detection. Early studies of diagnostic error focused on known cases that tended to represent the more harmful end of the spectrum, including closed malpractice claims [15] and cases identified through hospital quality assurance activities [9]. While these continue to be essential data sources for learning and exploring specific research questions [16], research has increasingly focused on developing systematic sampling strategies that improve the yield and timeliness of case detection.

#### Algorithmic (trigger-based) event detection

Healthcare operations generate vast data sets, and most modern electronic data warehouses can be searched to identify events of interest that might otherwise be undiscovered or underreported. Electronic triggers (e-triggers) are algorithms applied to the electronic health record (EHR) to identify patients with red-flag signals or symptoms who are at risk of misdiagnosis. E-triggers have been applied to identify diagnostic errors associated with specific care environments (e.g., unexpected return visits or care escalations in emergency departments [17]) and diseases (e.g., cancer [18]). E-trigger tools can allow health systems to monitor event rates, study contributing factors, and identify targets for improving diagnostic safety. Certain e-triggers can help detect diagnostic delays before they result in harm. Another approach is the Symptom-Disease Pair Analysis of Diagnostic Error (SPADE) method for large administrative data sets (i.e., billing, insurance claims) to map frequently missed diagnoses (e.g., stroke) to one or more previously documented high-risk symptoms (e.g., headache) [19]. One limitation is that algorithmic methods rely on data that may have inaccuracies or not provide a complete representation of clinical care.

#### Voluntary reports

Physicians are not always aware of their errors, but they are invaluable sources of insight into the errors they do discover. For instance, voluntary surveys of physicians have revealed that diagnostic errors occur frequently, even in common conditions [20], [21]. Healthcare organizations face steep obstacles to normalizing event reporting in routine practice [22]. Diagnostic errors are often still attributed to individual physicians’ skill or intellect, and thus are especially difficult to elicit through voluntary reporting [22], [23]. Physicians are more willing to bring attention to errors when reporting is easy, efficient, and aligned with a culture that prioritizes psychological safety. An intervention that encouraged physician reporting using reminders, scheduled reflection time, and monthly progress reports was successful in increasing reports of suspected diagnostic errors in a hospital medicine service [24]. Technological solutions, including mobile apps and EHR integration, have also been developed to facilitate clinician reporting [25].

#### Applying a diagnostic safety lens to other safety events

Diagnostic errors may occur as part of other safety events but are not recognized as such. For example, during care escalation events, a patient may be treated for an acute condition (i.e., shortness of breath), but failure to accurately identify the underlying cause (i.e., pneumonia vs. pulmonary embolus) may result in a diagnostic error. Since diagnostic errors may be obscured during complex events, investigators have used diagnostic safety measurement tools to enhance the investigation of events that may not have been initially classified as diagnosis related. Several resources have been introduced to improve data gathering, analysis, and learning from such safety events [26], [27].

#### Patient-reported events

Research studies have analyzed patients’ complaints [28] and used methods to capture patients’ perspectives related to their diagnosis [29]. Policies to give patients access to their medical records, now mandated by law in the U.S. [30], have opened new doors for patients to identify care discrepancies and errors in the medical record. These methods are limited in that patients and clinicians may differ in their understanding of what constitutes an error. Event reporting mechanisms for patients require further refinement, with a broader set of pathways for event investigation and adjudication.

### Measurement as a foundation for improvement

There is no perfect approach to identifying diagnostic events in practice, and a combination of measurement methods is preferable. Broadly, a prevailing approach is a two-stage event review and analysis process that combines at least one of the event detection strategies described above with a standardized chart review process [25]. In this approach, retrospective reviews help in learning about past events and encourage proactive remediation of care gaps. Methods to better analyze cases have also been developed in the past decade, with new diagnostic safety-specific tools and frameworks that can be used within traditional patient safety activities such as root cause analysis [26]. Guidance has been developed in the form of an AHRQ resource, “Measure Dx,” that helps translate measurement concepts from research into a pragmatic organizational strategy [25].

## Recommendations for future research

The field of diagnostic safety has evolved beyond only measuring diagnostic errors to studying the entire diagnostic process to understand how to achieve diagnostic excellence. In the following section, we highlight promising areas of research, describe some challenges that need to be overcome, and propose future directions to support research and accelerate its translation into practice.

### Emerging methods and approaches in diagnostic safety

#### Addressing measurement challenges

Further consensus is needed around a shared mental model and standardized definition of a diagnostic error and/or diagnostic safety event across the research enterprise as well as the application of tools to measure these events. For example, one area that could especially benefit from standardization is diagnostic safety event reporting. Implementation of reporting standards within healthcare organizations (e.g., for internal monitoring and quality improvement) and externally (e.g., reporting out through patient safety organizations) could accelerate diagnostic safety measurement and improvement activities. Additional areas for development include standards on the timeliness of diagnosis of certain conditions, especially those prone to being missed or delayed. Resilience engineering and Safety II, as applied to diagnosis, is also a nascent yet rapidly evolving area of study that will require standardized definitions for measuring individual and organizational resilience concepts relevant to the diagnostic process [31].

#### Patient co-development

Funding agencies and project sponsors can promote the engagement of diverse patients and families as partners in research and measurement activities by incentivizing projects that adopt co-design/co-development approaches while ensuring that funding is available to compensate patients and family partners. The use of participatory methods, such as the co-production of tools and processes and co-designing interventions, can enhance or facilitate patient and family engagement in diagnostic safety research and quality improvement initiatives.

#### Multidisciplinary perspectives in research

Given the sociotechnical complexities of diagnostic safety, multidisciplinary teams and approaches are needed to accelerate progress. Multidisciplinary research teams should aim to include additional disciplines beyond clinical medicine. For example, human factors experts enhance diagnostic safety research by addressing cognitive, environmental, and organizational elements that influence clinician decision-making and workflows, but few studies have applied rigorous human factors and cognitive science approaches to optimize interface designs or cognitive support to clinicians. Other disciplines that can substantially contribute to diagnostic safety work include health informatics, social sciences, cognitive science, and implementation science, among others.

#### Cross-cutting and disease-agnostic research

Many studies in diagnostic safety have aimed to address diagnostic concerns in specific high-risk conditions (e.g., cancer, sepsis, pulmonary embolism). Focusing on one disease or concepts such as the “Big 3” (e.g., cancer, cardiovascular, and infectious disease) leads to a disease-centered approach that is useful for the given condition but may limit generalizability to other conditions. For example, disease-focused approaches are likely to overlook important insights on cross-cutting issues, such as the management of diagnostic uncertainty in patients with undifferentiated symptoms. We recommend that, in addition to disease-focused approaches, future efforts focus on developing more disease-agnostic approaches that consider systems, processes, and cognitive issues that are cross-cutting and underlie most diagnostic errors. Adoption of cross-cutting methods and strategies can also help researchers and healthcare organizations apply these methods to understudied populations and respond more readily to patient-centered priorities or emerging system or population needs.

#### Use of sociotechnical approaches in the context of health information technology

A variety of sociotechnical interventions have been developed to help close the loop on clinician communication (e.g., orders, test results, and referrals). However, these interventions have been adopted slowly in real-world practice settings, and methods to support their implementation are needed. The body of research to study and improve system usability and address poorly designed EHR interfaces that burden clinicians and lead to diagnostic errors is also small. Novel methodologies for studying diagnostic decision-making in EHR-based environments include eye-tracking and computational ethnography (e.g., collecting digital trace data or metadata) that help better understand users’ true EHR interactions and information-seeking behaviors.

### Areas to prioritize for diagnostic safety research

In the following section, we propose five areas to prioritize for research, including cognitive science of diagnostic errors, costs related to diagnostic safety, interventions for patient engagement, artificial intelligence (AI) integration, and system-related interventions to improve diagnosis.

#### Cognitive science of diagnostic errors

Prior studies implicate cognitive contributing factors in most diagnostic errors. However, studies of cognitive processes in medicine have been conducted mainly in experimental or simulation settings. We suggest two main areas for future research on cognition and diagnostic decision-making. First, we need to understand how various elements of the clinical practice environment, such as documentation demands and EHR interface designs, affect clinicians’ cognitive load and diagnostic accuracy [32]. For instance, experimental studies suggest that negative shifts in effect (e.g., during a stressful encounter) may temporarily reduce cognitive resources devoted to clinical problem-solving [33], increasing the risk of error. Testing this and related hypotheses in real-world settings will require new experimental designs, which may incorporate methods such as wearable technologies or other unobtrusive observation methods to identify higher-risk states.

Second, we need to develop interventions to help clinicians become aware of their mistakes and devise strategies to remediate knowledge or reasoning gaps. For instance, experts have called for the development and implementation of systematic feedback on diagnostic performance to improve clinical reasoning and calibration [34]. However, clinicians currently have few incentives to incorporate these strategies. While individual clinicians are often motivated to become better diagnosticians, relatively few find it feasible to overcome the barriers of limited time, resources, and support to implement new diagnostic excellence practices. Health systems, professional societies, and state/federal agencies can provide these needed resources to ensure clinicians’ participation in diagnostic improvement activities. Furthermore, any addition to clinicians’ existing burden should be supported by strong evidence favoring the new practice and, ideally, accompanied by de-implementation of practices that do not benefit the clinician or patients.

#### Costs related to diagnostic safety and the “business case” for improving diagnosis

A report from the Organisation for Economic Co-operation and Development (OECD) estimated that 17.5 % of total healthcare expenditures are attributable to diagnostic errors and overdiagnosis [35]. However, estimates of the economic burden of diagnostic error are based on broad projections. Little is known about how healthcare financing, organization, and payment models affect diagnostic performance. Furthermore, at the organizational level, little is known about the return on investment for interventions to improve diagnosis. These knowledge gaps make it difficult to provide the “business case” for investing in diagnostic safety interventions. Moreover, there are few, if any, external incentives that encourage healthcare organizations to implement diagnostic safety interventions. The NASEM report suggested potential policy and payment levers to drive improvements in this area, including the recommendation that accrediting organizations (such as the Joint Commission) require healthcare organizations to systematically monitor, identify, and learn from diagnostic errors [5]. However, scientific progress in this area has been slow and is needed to inform policy and reforms. Efforts to revise payment models to incentivize diagnostic safety measurement and improvement activities and appropriately reimburse the cognitive effort and teamwork are required to achieve diagnostic safety [36]. Furthermore, better methods are needed to quantify costs related to the impact of diagnostic error and of strategies to mitigate related harms.

#### Interventions for patient engagement

More effective partnerships with patients are needed to design and implement interventions to improve and monitor diagnostic safety. In clinical practice settings, strategies to improve communication and engagement during the diagnostic encounter are relevant and highly valued by patients but remain underused. Patients are also essential but often overlooked partners in the design and testing of patient-facing interfaces (e.g., patient portals) and reports to communicate diagnostic findings [37]. Finally, a variety of strategies exist to guide patient engagement in clinical encounters, research, and system improvement, and would benefit from implementation efforts. Future efforts should focus on engagement of patients who are more at risk of being disempowered, including older adults, patients with limited English proficiency, and patients with disabilities.

#### Artificial intelligence integration

Artificial intelligence (AI) has the potential to transform not only how individual clinicians and healthcare teams diagnose patients [38], but also how the diagnostic process and diagnostic errors themselves are measured [39]. AI-based tools to support diagnosis have varying evidence of validity, effectiveness, and clinical feasibility. For instance, while significant advances in AI have improved image interpretation in pathology and radiology, the role of generative AI in enhancing clinical diagnosis in real-world practice is still evolving. Rigorous research is needed to understand how clinicians interact with generative AI to arrive at a diagnosis, the risks and benefits of using AI for this purpose, how AI should be tailored to different settings and expertise, and patients’ and families’ perspectives on use of these AI tools. Moreover, as the use of AI in medicine becomes more ubiquitous, vigilance is needed to identify and study its use for tasks that can indirectly affect the diagnostic process. For example, physicians have raised concerns that the use of ambient AI scribes to create clinical notes may have the unwanted effect of taking away opportunities for deeper reflection that can lead to better diagnostic decisions [40]. Aside from studying the impact of AI-based interventions on diagnosis, pragmatic frameworks must also be developed to guide the implementation and evaluation of AI tools for diagnosis [41].

#### System-related interventions to improve diagnosis

Implementation of dedicated programs and activities to address diagnostic error remains uncommon in healthcare organizations. Diagnostic safety activities have been described in the context of existing quality and safety frameworks, such as learning health systems [42]. Additionally, broadly generalizable principles and recommendations have now emerged and are summarized in recent publicly available resources to support implementation of diagnostic safety practices. For example, to build national consensus on recommended practices for hospitals to improve diagnosis, the Leapfrog Group assembled a report describing 29 evidence-based practices that hospitals can use to prevent diagnostic harm to patients [43]. These types of recommended organizational structures and practices are only beginning to be evaluated systematically. Additional guidance has also been developed, such as the Safer Dx Checklist, which helps conduct a systematic organizational assessment of evidence-based safety practices [44].

## Conclusions and next steps

The field of diagnostic safety research has grown from a handful of small research programs to an international, multidisciplinary community. In comparison to research from a decade ago or longer, more studies are now being conducted in real-world healthcare settings, a reflection of efforts to begin to bridge the research-practice gap. Concurrently, the number of collaborative and multi-institutional studies is growing, which may help build consensus around standard definitions and procedures for future research. New data sources have enhanced our understanding of diagnostic errors, with both patient engagement and increasing use of health information technology to enhance discovery and learning.

While research has led to progress in diagnostic safety over the last decade, fundamental gaps remain in both the quality and quantity of research needed to make further advances. However, the science is now robust enough that all healthcare organizations, clinicians, and patients can start to take steps to reduce diagnostic errors. Some healthcare organizations have committed resources to improving diagnosis [45]. As more systems follow the lead of these pioneering organizations, and as external advisory groups provide new guidance on diagnostic excellence, both research and implementation gaps remain to be filled. Efforts must be accelerated to monitor healthcare organizations’ implementation of resources and guidance to reduce diagnostic errors and to measure the downstream impact on patient outcomes and clinician well-being. To encourage the uptake of new diagnostic safety improvement approaches, multicenter learning collaboratives could promote the translation of diagnostic work into routine clinical practice. New programs and policies that incentivize clinicians and health care organizations to engage in diagnostic safety work should be developed.

Research impact must go beyond the traditional metrics of publications. More meaningful research impact includes influence on practice and policy as well as the development and implementation of pragmatic tools, products, or innovations that are subsequently used in real-world settings. To promote broad-scale improvement in diagnostic safety, we have provided several next steps and recommendations for various audiences, including researchers, research funders, safety professionals, and policymakers, who plan to conduct or support research and implementation activities aimed at reducing preventable diagnostic harm over the next decade.
